# Electrophysiological characteristics in adolescents with non-suicidal self-injury: an event-related potential study and source analysis

**DOI:** 10.3389/fpsyt.2025.1596035

**Published:** 2025-08-07

**Authors:** Sung-Hoon Yoon, Ji Sun Kim, Hyeon-Ah Lee, Woo-Seung Lee, Young Wook Song, Se-Hoon Shim

**Affiliations:** ^1^ Department of Psychiatry, Wonkwang University Hospital, Iksan, Republic of Korea; ^2^ Department of Psychiatry, Soonchunhyang University Cheonan Hospital, Cheonan, Republic of Korea; ^3^ Department of Applied Artificial Intelligence, Hanyang University, Ansan, Republic of Korea

**Keywords:** interpersonal relations, non-suicidal self-injury, event related potentials, electroencephalography, depression

## Abstract

**Introduction:**

Non-suicidal self-injury (NSSI) is a serious concern in adolescents and is associated with impairments in impulsivity and social functioning. However, the underlying neural mechanisms remain unclear. This study aimed to examine inhibitory control and its association with depressive symptoms and interpersonal distress in adolescents with NSSI using event-related potentials (ERPs) and source-level analysis.

**Methods:**

A total of 51 adolescents with NSSI and 50 HC were recruited. Psychological characteristics were assessed using standardized scales including the Interpersonal Needs Questionnaire (INQ) and Short UPPS-P Impulsivity Scale (SUPPS-P). EEG were recorded during a go/no-go task to measure P3 amplitudes. Source analysis was performed to localize the neural activity. Group differences were analyzed using RMANOVA, followed by Pearson correlation and mediation analyses to evaluate the relationships among the variables.

**Results:**

The NSSI group showed significantly lower accuracy than HCs. The interaction between group and electrode site was significant (p = .032, ηp² = .010), indicating spatially specific reductions in no-go P3 amplitude in the NSSI group. No-go P3 at Fz was negatively correlated with depressive symptoms and interpersonal distress. Source-level analysis revealed marginally reduced activation in the right superior frontal gyrus in the NSSI group but diminished after adjusting for depressive symptoms. Mediation analysis indicated that depressive symptoms significantly mediated the relationship between both neural indices and interpersonal distress.

**Conclusions:**

Adolescents with NSSI exhibit impaired inhibitory control that is associated with depressive symptoms and social distress. These findings highlight the role of affective dysregulation in linking cognitive control deficits to interpersonal functioning in NSSI.

## 1 Introduction

Non-suicidal self-injury (NSSI), defined as the direct and deliberate destruction of one’s own bodily tissue in the absence of suicidal intent, has recently increased among young people, raising significant alarm among psychiatric researchers (1, 2). This behavior encompasses a range of self-inflicted actions such as cutting, burning, or hitting oneself, and is primarily used as a maladaptive coping mechanism to relieve emotional distress or regulate emotional dysregulation (3).

Although NSSI is defined by the absence of suicidal intent, its clinical significance should not be underestimated, as it is strongly associated with elevated risks of suicidal ideation and attempts (4, 5). These findings highlight that NSSI is not a benign behavior, but rather a marker of severe emotional distress, interpersonal dysfunction, and frequent psychiatric comorbidities such as depression and anxiety (6). These findings emphasize the need for early identification and a deeper understanding of underlying mechanisms to inform effective prevention strategies.

Theoretical models suggest that NSSI is driven by multiple, interacting psychological processes. One prominent framework is the Interpersonal-Psychological Theory of Suicide (IPTS), which proposes that perceived burdensomeness (PB) and thwarted belongingness (TB) increase the risk of self-injurious thoughts and behaviors (7). PB, defined as the perception of being a burden to others and being worth more dead than alive, and TB, defined as a feeling of disconnection from others (8), are key components of the IPTS. Recent studies have suggested a relationship between interpersonal distress and NSSI among adolescents (9–11). Adolescents who report greater interpersonal distress are more likely to engage in self-injury as a maladaptive coping mechanism to alleviate emotional pain or seek social connection (11). Therefore, interventions based on the IPTS are suggested to improve the symptoms of NSSI in adolescence.

In addition to interpersonal difficulties, deficits in inhibitory control have been widely implicated in the emergence and maintenance of NSSI behaviors. Adolescents engaging in NSSI often exhibit heightened impulsivity and difficulty regulating their actions in response to emotionally charged situations (12). Neurocognitive studies using event-related potentials (ERPs) point to the no-go P3 as a fronto-cingulate marker of conflict monitoring and response suppression. This component—and thus the capacity to allocate attentional and control resources under changing demands—has been shown to be attenuated in both impulsive and affective disorder (13, 14). Studies have shown that individuals with NSSI exhibit altered brain activity patterns, such as reduced no-go P3 amplitudes, which are associated with impaired cognitive control and increased impulsivity (15).

Furthermore, depressive symptoms are not only prevalent among adolescents with NSSI, but are also significantly associated with impairments in inhibitory control (16, 17). Individuals with depressive mood demonstrate attenuated no-go P3 amplitudes and longer no-go reaction times in the go/no-go task, suggesting deficits in cognitive control processes (18–20). In addition, depressive symptomatology has been shown to correlate with increased interpersonal distress (21). These findings align with theoretical perspectives suggesting that depressed mood may bridge the gap between impaired inhibitory control and social-affective impairments. However, empirical research directly testing such mediation models in NSSI populations remains scarce.

Although several studies have documented altered ERP responses in populations with NSSI, the direct relationship between neural markers of inhibitory control and interpersonal distress which are measured by psychological scales has rarely been examined. Understanding how these psychological and neurophysiological factors interact may provide critical insights into the mechanisms underlying NSSI and inform targeted interventions. Therefore, the present study aimed to investigate the cognitive and neural correlates of NSSI among adolescents, focusing on both psychological and electrophysiological indices. Addressing this gap, the present study examined both psychological characteristics, including depression and interpersonal distress, and electrophysiological features represented inhibitory control in adolescents with NSSI.

We hypothesized that no-go P3 amplitude at the midline would be negatively associated with interpersonal distress. Furthermore, we proposed that depressive symptoms would mediate this association, such that reduced P3 amplitude would be linked to heightened interpersonal distress indirectly through elevated depressive symptoms.

To strengthen anatomical inferences, we additionally performed source-level analyses to identify cortical generators supporting inhibitory control during the no-go task.

## 2 Materials and methods

### 2.1 Participants

Altogether, 50 adolescents with NSSI and 51 HC were enrolled between January 2020 and May 2023. All patients were between the ages of 12 and 19 years and right-handed, with normal hearing ability. All participants were recruited from outpatient psychiatric clinics at Soonchunhyang University Hospital via clinician referral. Participants were interviewed using the Korean version of the MINI International Neuropsychiatric Interview. All adolescents in the NSSI group met diagnostic criteria for Major Depressive Disorder (MDD) as their primary diagnosis, based on the MINI International Neuropsychiatric Interview. Participants with bipolar disorders, psychotic disorders, neurodevelopmental disorders such as intellectual disabilities and autism spectrum disorder, neurological or severe medical diseases, a history of alcohol or substance abuse/dependence, head trauma, or who were currently pregnant were excluded from the study through screening interviews. In the case of NSSI, EEG was performed when the patients were drug naïve. Only participants who did not take psychiatric drugs were recruited, and EEG was performed during their first visit. This study was approved by the Institutional Review Board and Ethics Committee of Soonchunhyang University Cheonan Hospital, and all experimental protocols were approved by the committee (2020-07-042). Participants were informed that they could end the study at any time if they wanted to, and the study was performed in accordance with approved guidelines. Informed consent was obtained from all study participants, and all consent forms were completed by the participants and their parents.

### 2.2 Clinical measures

All participants were assessed for psychiatric symptoms such as depressive mood, anxiety, impulsivity, and emotional dysregulation. To assess the clinical characteristics of the abovementioned psychiatric symptoms, the Center for Epidemiologic Studies Depression scale (CES-D), State-Trait Anxiety Inventory (STAI), Short version of UPPS-P impulsive behavior scale (SUPPS-P), Acquired Capability for Suicide Scale (ACSS), Childhood Trauma Questionnaire (CTQ), Interpersonal Needs Questionnaire (INQ), Difficulties in Emotion Regulating Scale-16 (DERS-16) and Pain Catastrophizing Scale (PCS) were administered. The CES-D is a 20-item self-report scale designed to measure depressive symptoms over the past week, with each question scored on a scale of 0 to 3 points. Higher scores are positively correlated with severe depression. We used the Korean version of the CES-D, which has been validated for Korean adolescents (22). The STAI distinguishes between state anxiety, which is a temporary emotional state, and trait anxiety, which is defined as the general tendency to experience anxiety. Each item is scored on a 4-point Likert scale, with higher scores indicating greater levels of anxiety. The SUPPS-P measures impulsivity across five dimensions: negative urgency, lack of perseverance, lack of premeditation, sensation seeking, and positive urgency (23). All items are scored on a Likert scale from 1 to 4. The ACSS is a 20-item self-reported measure of the extent to which individuals perceive themselves as capable of performing or being exposed to potentially dangerous or fatal situations, including suicide, with scores ranging from 0 to 4 (24). We also used the CTQ to measure the patients’ traumatic experiences during childhood. The CTQ is a self-reported scale that defined as 5 different types of childhood abuse and neglect, which are rated on a scale from 1 to 5 (25). The INQ is a self-reported tool designed to assess two constructs central to the IPTS: TB and PB (26). It consists of 15 items rated on a 7-point Likert scale ranging from 1 to 7. Higher total scores on each subscale indicate greater levels of PB or TB. The DERS-16 is a self-report tool that consists of 16 items, with each item’s score ranging from 1 to 5, higher score means participants experience more emotional dysregulation (27). Finally, we used the PCS, a 13-item self-report scale ranged from 0 to 5, to measure the tendency to magnify and ruminate about pain experiences, often linked to emotional dysregulation and self-injurious behaviors (28). And also, to assess the characteristics and motivations of NSSI, we used the Korean version of the Inventory of Statements About Self-Injury (K-ISAS). The K-ISAS consists of two sections: Section 1 assesses the lifetime frequency and types of NSSI behaviors, and Section 2 evaluates 13 functional motivations for NSSI, including affect regulation, self-punishment, anti-dissociation, anti-suicide, interpersonal influence, interpersonal boundaries, sensation seeking, peer bonding, marking distress, toughness, autonomy, revenge, and self-care, through 39 items rated on a 3-point Likert scale (29). In this study, we examined both the behavioral patterns and functional subscales of NSSI.

### 2.3 EEG data acquisition and analysis

EEG data were collected while the participants were seated approximately 60 cm away from a computer monitor in a soundproof EEG room. Signals were recorded using a NeuroScan SynAmps amplifier (Compumedics USA, El Paso, TX, USA) with 64 Ag/AgCl electrodes positioned on a QuickCap adhering to the extended 10–20 electrode placement system. Key electrodes were positioned at the frontal (Fz), central (Cz), and parietal (Pz) sites, with the Earth electrode at FPz. Electrodes for monitoring eye movements were placed infraorbitally and mastoid electrodes were used as references. The impedance was maintained below 10 kΩ throughout the sessions. EEG signals were band-pass filtered (0.1–100 Hz) and sampled at 1000 Hz. The CURRY 8 software (Compumedics USA, Charlotte, NC, USA) was used for preprocessing. Large artifacts, such as those caused by muscle movements or gross body shifts, were visually inspected and manually excluded by a licensed clinical neurophysiology technologist who was blinded to participants’ group assignments Eye movement-related artifacts were corrected using automated preprocessing methods. Independent component analysis (ICA) was subsequently performed using CURRY 8 to identify and remove artifacts. Following artifact correction, the EEG data were re-referenced to a reference electrode standardization technique (REST) to achieve a more neutral reference (30). The data were further band-pass filtered (1.0–30 Hz) and segmented into epochs from 500 ms pre-stimulus to 900 ms post-stimulus. Baseline corrections were performed at pre-stimulus intervals. Epochs with residual artifacts exceeding ±75 µV at any electrode site were excluded from further analysis. Only clean epochs were averaged across trials and participants for event-related potential (ERP) analysis. Based on prior research identifying no-go ERPs as indicators of behavioral inhibition (15), this study focused on no-go trials for ERP analyses.

### 2.4 Behavioral task paradigm

An auditory go/no-go task using an oddball paradigm was employed to elicit ERPs. Participants wore headphones and were instructed to press the spacebar as quickly and accurately as possible in response to the target tone (go condition) and to withhold responses to the non-target tone (no-go condition). A total of 400 trials were presented, comprising 85% go trials and 15% no-go trials. The target tone (no-go) was 1,500 Hz, while the non-target tone (go) was 1,000 Hz, with a 1,500 ms inter-trial interval. Stimuli were generated using E-Prime software (Psychology Software Tools; Pittsburgh, PA, USA). This study analyzed the P300 ERP components (the most positive peak between 250 and 500 ms post-stimulus, P3) at the frontal (Fz), fronto-central (FCz), central (Cz), and parietal (Pz) electrodes. The time windows for the analysis were based on previous studies (20). Behavioral data, including go accuracy, no-go accuracy, and reaction times were collected using E-Prime software.

### 2.5 Statistical analysis

Group differences in demographic characteristics, clinical measures, and behavioral task performance were analyzed using the chi-square test for categorical variables and independent t-tests for continuous variables, following the verification of normality assumptions. For behavioral task, go accuracy, no-go accuracy, and go reaction time were compared between groups using independent *t*-tests. To account for three parallel comparisons, Benjamini-Hochberge false discovery rate (FDR) was applied. For ERP data, repeated-measures analysis of variance (RMANOVA) was performed on no-go P3 amplitudes across midline electrode sites (Fz, FCz, Cz, and Pz), with electrode as a within-subject factor and group (NSSI vs. HC) as a between-subject factor, CES-D entered as a covariate. After conducting RMANOVA, we performed Pearson correlation analyses to examine the associations between no-go P3 amplitude at frontal electrode sites (Fz and FCz) and psychological variables, including depressive symptom severity (CES-D scores) and interpersonal distress (INQ scores). Based on extensive prior literature implicating frontal midline sites in inhibitory control measured by no-go p3, *a priori* follow-up correlation analysis was restricted to Fz and FCz (31, 32) and FDR was used for adjust p-value. Statistical significance was defined as p ≤ 0.05. All analyses were performed using SPSS version 26.0 (SPSS Inc., Chicago, IL, USA) and R statistical software.

### 2.6 Source analysis

Source analysis was conducted to identify the neural generators of the no-go P3 component using the Brainstorm toolbox (33). A depth-weighted L2 norm solution was applied to estimate cortical current density time series. The forward model was computed using a three-layer boundary element model (BEM) based on the MNI/Colin27 anatomy template. Source activity was estimated over 15,002 cortical vertices, and cortical regions were defined using the Desikan–Killiany atlas, which partitions the cortex into 34 anatomical regions per hemisphere. For each region of interest (ROI), representative values were obtained by applying principal component analysis (PCA) to source signals within a 5 mm radius of each ROI centroid (34). Based on prior literature linking fronto-cingulate and parietal regions to inhibitory control and affective processing, a subset of midline and adjacent ROIs was selected for group comparisons. These included the bilateral rostral and caudal anterior cingulate cortices, posterior cingulate cortices, medial orbitofrontal cortices, superior frontal gyri, and paracentral lobules (35, 36). To complement the frequentist approach and provide a graded measure of evidence for or against group differences, Bayesian t-tests were also performed using the BayesFactor package in R (37). Because we tested a large number of *a priori* ROIs, conventional family-wise error corrections risk masking genuine effect trends. Instead, we used Bayesian independent-samples t-tests to quantify evidence directly via Bayes factors, which obviate the need for *ad-hoc* α-level adjustments while preserving sensitivity to meaningful patterns (38). BF_10_ values greater than 3 were interpreted as moderate evidence for the alternative hypothesis, while values below 1/3 were taken as moderate evidence for the null.

### 2.7 Mediation analysis

To examine potential mediation effects, a causal mediation analysis was conducted using the R package mediation (39). The model tested whether depressive symptoms mediated the relationship between no=go p3 activity and Interpersonal needs (INQ). Two linear models were specified: one predicting CES-D from Fz amplitude, and the other predicting INQ from both CES-D and Fz amplitude. The indirect (mediation) and direct effects were estimated using nonparametric bootstrapping with 1,000 simulations to generate percentile-based confidence intervals.

## 3 Results

### 3.1 Participants

**Figure 1:**
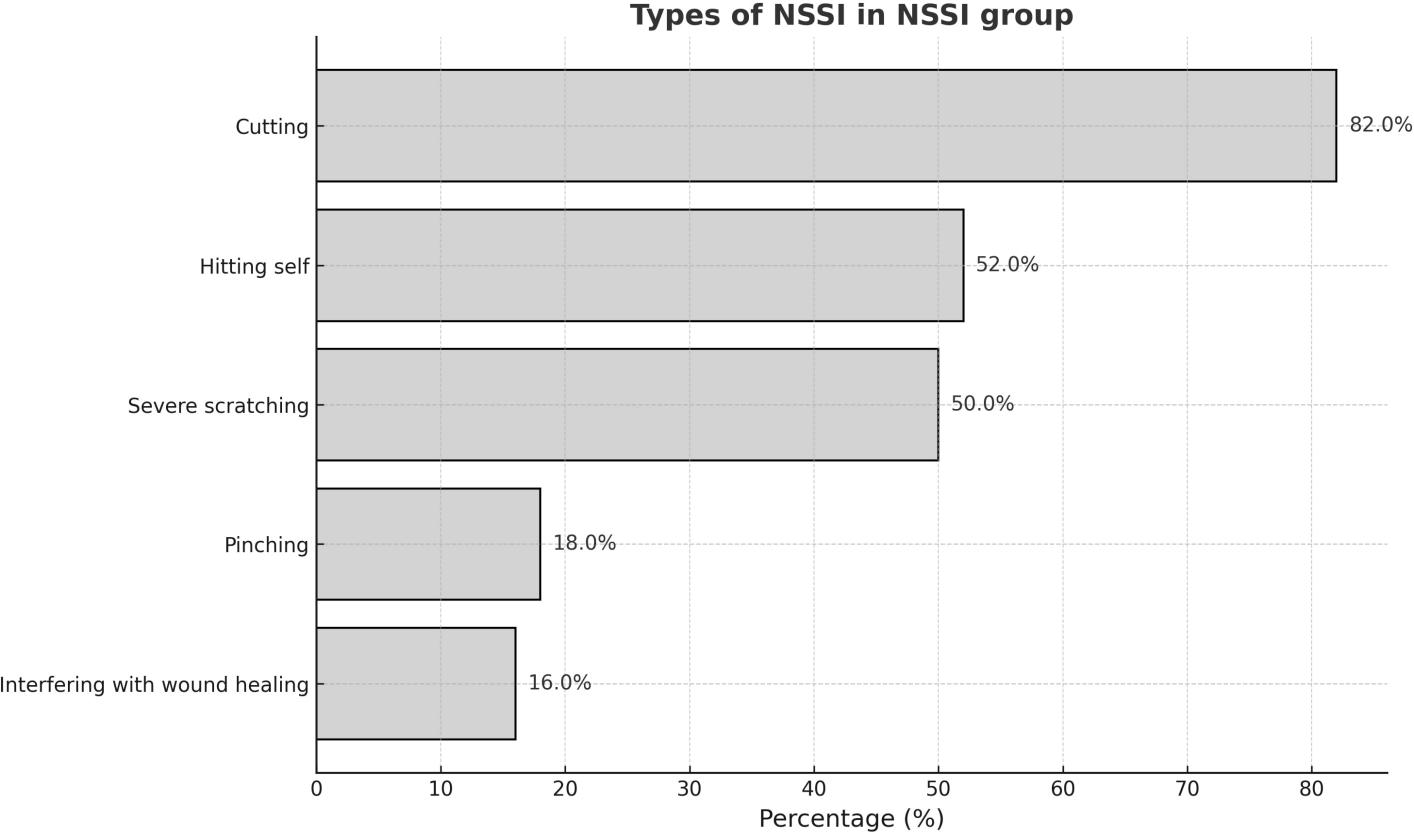
Prevalence of NSSI behavior in NSSI groups.

All adolescents in the NSSI group met diagnostic criteria for Major Depressive Disorder (MDD) according to the MINI. **Table 1** presents the demographic data and clinical measurements of all participant groups. No significant differences in age (p = 0.074), sex (p =0.104), or educational level (p = 0.211) were observed between the two groups. The NSSI group had significantly higher scores on all psychological scales than the HC group, except for the PCS (p = 0.208). **Table 2** shows that after adjusting for CES-D and STAI scores, only ACSS and INQ scores were significantly different between the NSSI and HC groups (p < 0.001). In the NSSI group, cutting was the most reported self-injurious behavior, endorsed by 82.0% of participants (**Figure 1**). This was followed by hitting oneself (52.0%) and severe scratching (50.0%). Less frequently endorsed behaviors included pinching (18.0%) and interfering with wound healing (16.0%). From the perspective of motivation of NSSI, affect regulation, self-punishment, and anti-dissociation were the most highly endorsed NSSI functions based on responses to the K-ISAS Section 2.

**Table 1:** Comparison of demographics and psychological test results between NSSI and HC groups.

Variable		NSSI (50) (Mean ± SD)	HC (51) (Mean ± SD)	t/χ²	p value
Age		15.77 ± 1.68	15.19 ± 1.56	1.804	0.074
Sex	Male	12 (24%)	21 (41.18%)	2.65	0.104
	Female	38 (76%)	30(58.82%)		
Education		7.62 ± 1.84	7.122.16	1.26	0.211
CES-D		43.28 ± 13.06	16.63 ± 7.77	12.016	<0.001*
State-anxiety		63.06 ± 11.16	37.16 ± 9.71	12.452	<0.001*
Trait-anxiety		66.14 ± 9.88	38.45 ± 11.34	13.073	<0.001*
DERS16		58.38 ± 14.86	33.30 ± 13.65	8.587	<0.001*
SUPPSP		45.00 ± 10.32	40.15 ± 8.50	6.230	<0.001*
CTQ		50.32 ± 12.10	45.60 ± 9.78	5.876	<0.001*
PCS		42.15 ± 9.45	36.45 ± 7.91	4.560	0.208
ACSS		39.32 ± 8.20	34.90 ± 7.54	5.670	<0.001*
INQ		44.78 ± 10.03	38.50 ± 9.12	6.221	<0.001*

SD, standard deviation; CES-D, Center for Epidemiologic Studies Depression Scale; DERS16, Difficulties in Emotional Regulation Scale-16; SUPPSP, Short version of the UPPS-P; CTQ, Childhood Trauma Questionnaire; PCS, Pain Catastrophizing Scale; ACSS, Acquired Capability for Suicide Scale; INQ, Interpersonal Needs Questionnaire.

**Table 2:** Comparison of psychological test results between NSSI and HC groups after adjusting CES-D and STAI score.

Variable	NSSI (Mean ± SD)	HC (Mean ± SD)	F-value	p-value
DERS16	58.38 ± 14.86	33.3 ± 13.65	2.188	0.143
UPPSPSF	50.9 ± 10.02	40.39 ± 7.39	0.688	0.409
CTQ	65.6 ± 18.31	40.37 ± 9.6	9.611	0.003
PCS	26.94 ± 14.66	23.8 ± 8.47	1.252	0.266
ACSS	45.8 ± 17.78	4.28 ± 10.88	63.521	<0.001
INQ	54.8 ± 11.25	18.0 ± 14.62	17.273	<0.001

SD, standard deviation; CES-D, Center for Epidemiologic Studies Depression Scale; STAI, State-Trait Anxiety Inventory; DERS16, Difficulties in Emotional Regulation Scale-16; SUPPSP, Short version of the UPPS-P impulsive behavior scale; CTQ, Childhood Trauma Questionnaire; PCS, Pain Catastrophizing Scale; ACSS, Acquired Capability for Suicide Scale; INQ, Interpersonal Needs Questionnaire.

### 3.2 ERP

#### 3.2.1 Behavioral outcomes

**Table 3** presents the behavioral outcomes of the no-go paradigm. Compared to the HC, NSSI group showed significantly lower accuracy on both go trials (t (96) = 4.26, *p* <.001) and no-go trials (t (96) = 5.24, *p* < .001). Reaction times were longer in the NSSI group than in the HC group, though this difference did not reach statistical significance (t (96) = 1.85, 0.68).

**Table 3:** Behavioral performance in the go/no-go task for adolescents with non-suicidal self-injury (NSSI) and healthy controls (HC).

Variable	NSSI (Mean ± SD)	HC (Mean ± SD)	t	p value
Go accuracy (%)	95.0 ± 8.0	99.68 ± 0.73	4.26	<0.001*
Nogo accuracy (%)	88.0 ± 14.0	98.00 ± 3.00	5.24	<0.001*
Reaction time (ms)	432.29 ± 78.23	401.17 ± 89.97	1.85	0.068

NSSI, non-suicidal self-injury; HC, Healthy control; SD, standard deviation *means p-value < 0.001.

#### 3.2.2 Amplitude and latency

A repeated-measures ANCOVA was conducted on no-go P3 amplitudes with Group (NSSI vs. HC) as a between-subjects factor, Electrode (Fz, FCz, Cz, Pz) as a within-subjects factor, and CES-D score as a covariate. There was a significant main effect of Electrode emerged (F (3, 297) = 8.39, p <.001, η_p_² = .078). Crucially, the Group × Electrode interaction was also significant (F (3, 297) = 2.88, p = .0361, η_p_² = .010) indicating that the pattern of group differences varied across electrode sites (**Table 4**). Although there was no overall main effect of Group on no-go P3 amplitude, the significant Group × Electrode interaction indicates that group differences depend on electrode site. RMANOVA showed no significant main effect of group on no-go P3 latency (*F* (1, 98) = 0.02, *p* = .89).

**Table 4:** No-go P3 Amplitude: Repeated-Measures ANOVA Results, CES-D as covariates.

Effect	df	F	P value	ηp²
Group	1, 98	3.13	0.079	0.031
Electrode	3, 297	8.39	<0.001**	0.012
CES-D	1, 98	1.19	0.269	0.078
Group * Electrode	3, 297	2.88	0.032*	0.010

CES-D, Center for epidemiologic studies for depression scale, *means p-value < 0.05, **means p-value<o.001.

#### 3.2.3 Source analysis

Bayesian model comparison indicated that most frontal regions favored the null model (BF_10_ < 1), only the Rt. superior frontal gyrus showed a BF_10_ > 1 (BF_10_ ≈ 1.38), indicating weak evidence favoring group differences in that ROI. NSSI group showed decreased brain activity in Rt. superior frontal gyrus. However, after adjusting for depressive symptoms, the evidence for group differences substantially decreased across all ROIs. In particular, the BF_10_ for the right superior frontal gyrus dropped to 0.33 (**Table 5**).

**Table 5:** Bayesian model comparison (BF_10_) for group differences with and without CES-D as covariate.

Brain Region	BF_10_ (No Covariate)	BF_10_ (With CES-D)
Rostral anterior cingulate (L)	0.815	0.056
Rostral anterior cingulate (R)	0.211	0.001
Medial orbiotofrontal gyrus (L)	0.229	0.004
Medial orbitofrontal gyrus (R)	0.255	0.002
Superior frontal gyrus (L)	0.213	0.005
Superior frontal gyrus (R)	1.380*	**0.327**

BF_10_ values greater than 1 indicate evidence in favor of group differences (alternative hypothesis), while values less than 1 favor the null model. * highlight the only region that initially favored the alternative model, which diminished after adjusting for depressive symptoms.

### 3.3 Correlations

In the full sample, the total score of CES-D was positively correlated with those of INQ, indicating higher depressive symptoms are closely linked to greater interpersonal distress. Within the NSSI group, affective regulation was positively correlated with CES-D (r = 0.27, p < 0.001, q < 0.001) and with INQ (r = 0.26, p <0.001, q < 0.001). Self-Punishment showed weaker but still significant correlations with CES-D (r = 0.15, p = 0.031, q = 0.031) and INQ (r = 0.18, p = 0.009, q = 0.011).

#### 3.3.1 Scalp-level ERP with psychological scales

**Figure 2:**
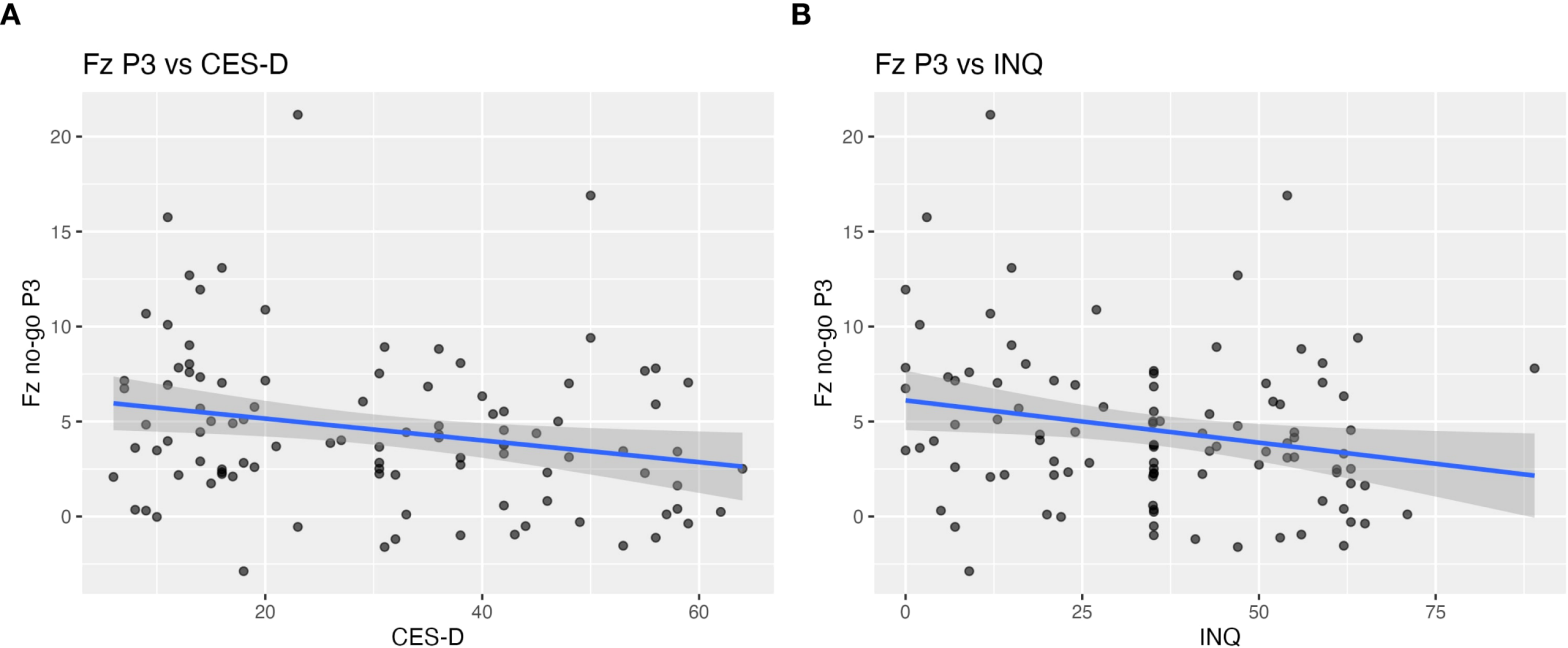
Scalp-level correlations between Fz no-go P3 amplitude and clinical measures. Scatter plots illustrating the relationship between frontal no-go P3 amplitude at Fz and **(A)** depressive symptoms (CES-D) and **(B)** interpersonal distress (INQ) across all participants. Reduced P3 amplitude at Fz was moderately associated with higher CES-D scores (r = –0.23, p = 0.019, q = 0.048) and higher INQ scores (r = –0.22, p = 0.024, q = 0.048).

No-go P3 at Fz showed moderate negative correlations with both depressive symptoms (CES-D: *r* = –0.23, *p* = 0.019, *q* = 0.048) and interpersonal distress (INQ: *r* = –0.22, *p* = 0.024, *q* = 0.048) after FDR correction (**Figure 2**). Correlation between No-go P3 at FCz and psychological scales did not reach significance after FDR correction (CES-D: *r* = –0.21, *p* = 0.040, *q* = 0.080; INQ: *r* = –0.18, *p* = 0.072, *q* = 0.144).

#### 3.3.2 Source-level ROI with psychological scales and ISAS

**Figure 3:**
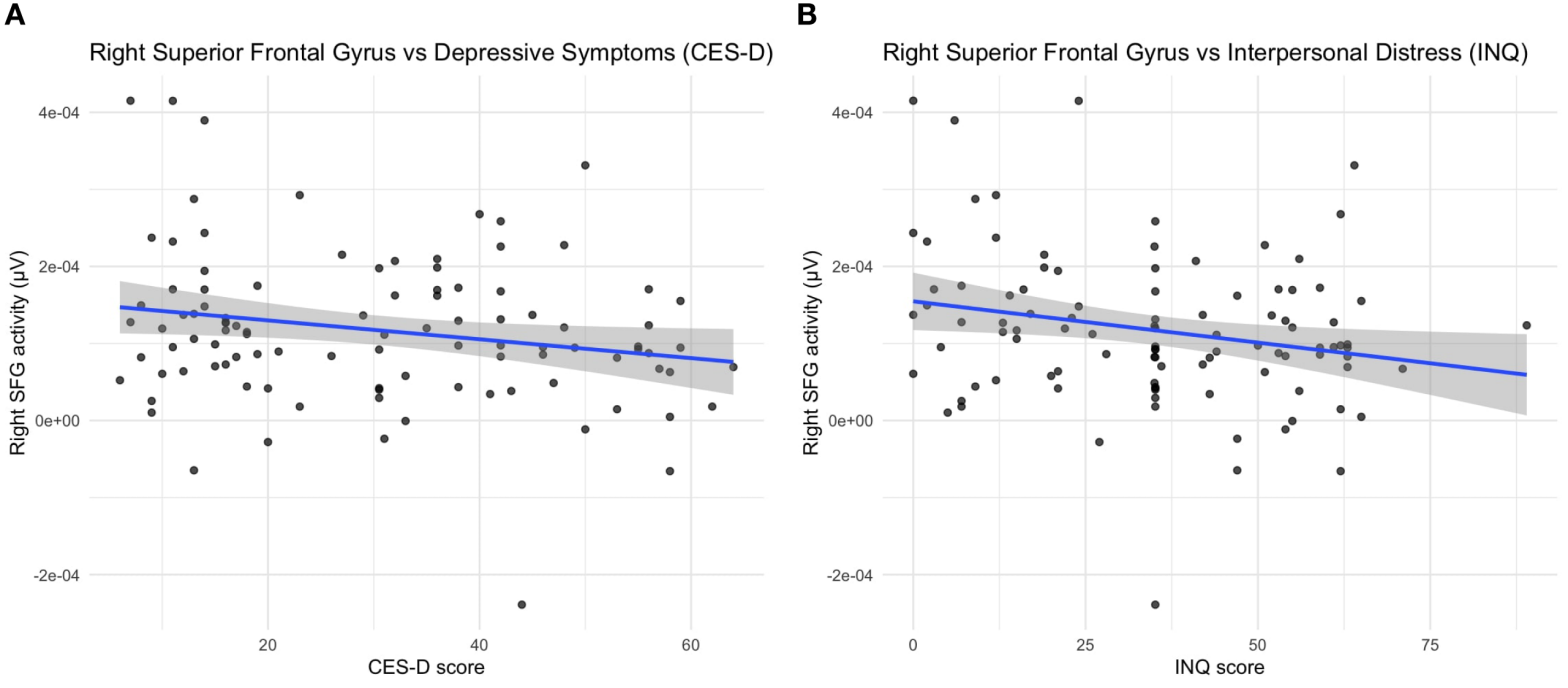
Source-level correlations between right superior frontal gyrus activity and clinical measures. Scatter plots illustrating the relationship between right superior frontal gyrus (SFG) source activity and **(A)** depressive symptoms (CES-D) and **(B)** interpersonal distress (INQ) across all participants. Reduced SFG activity was moderately associated with higher CES-D scores (r = –0.21, p = 0.036, q = 0.036) and higher INQ scores (r = –0.23, p = 0.022, q = 0.036).

Right superior frontal gyrus (SFG) source activity showed negative correlations with depressive symptoms (CES-D: r = –0.21, p = 0.036, q = 0.036) and with interpersonal distress (INQ: r = –0.23, p = 0.022, q = 0.036) after FDR correction (**Figure 3**). Within the NSSI group, SFG activity was marginally associated with affective regulation (r = –0.25, p = 0.081, q = 0.162) but not with self-punishment (r = –0.04, p = 0.66, q = 0.66).

### 3.4 Mediation analysis

To examine whether depressive symptoms mediated the relationship between inhibitory control and interpersonal distress, a mediation analysis was conducted with Fz no-go P3 amplitude as the predictor, CES-D as the mediator, and INQ as the outcome.

**Figure 4:**
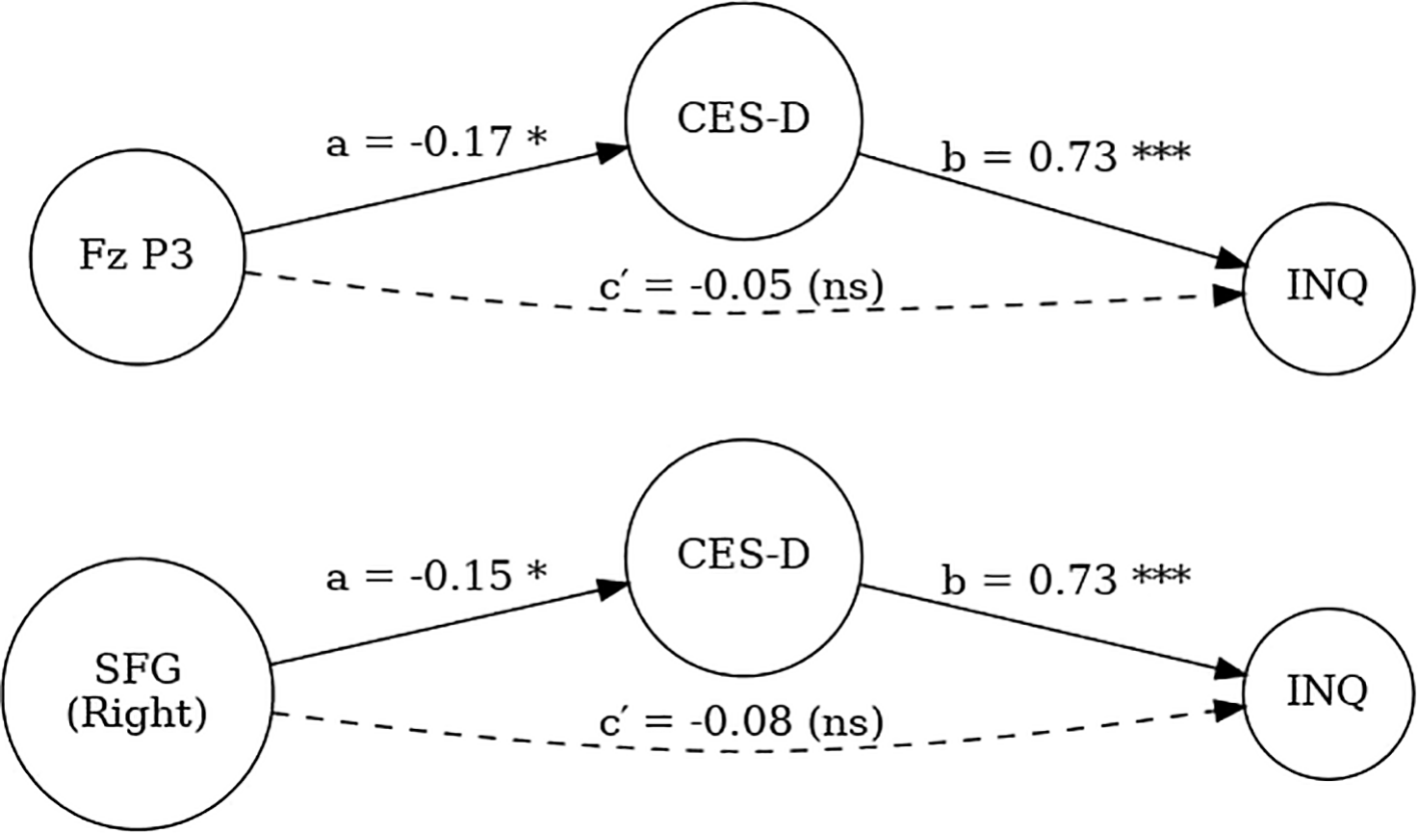
Mediation models testing the indirect effects of Fz no-go P3 amplitude (top) and right superior frontal gyrus (SFG) activity (bottom) on interpersonal distress (INQ) through depressive symptoms (CES-D). Path *a* represents the effect of neural indices on CES-D, path *b* represents the effect of CES-D on INQ, and path *ca* represents the direct effect of neural indices on INQ after accounting for CES-D. Indirect effects were statistically significant, while direct effects were non-significant. (*p* <.05*, **p <.001, ns = not significant).

The indirect effect was statistically significant (ACME = -0.17, 95% CI [-0.32, -0.03], p = .018), whereas the direct effect was non-significant (ADE = -0.05, p = .474). The total effect remained significant (p = .040), with approximately 76% of the effect of Fz no-go P3 on INQ being mediated through depressive symptoms (proportion mediated = 0.76, p = .042). Similarly, a mediation analysis was performed using right superior frontal gyrus (SFG) source activity as the predictor, CES-D as the mediator, and INQ as the outcome. The indirect effect was also significant (ACME = -0.15, 95% CI [-0.28, -0.01], p = .030), while the direct effect was not significant (ADE = -0.08, p = .216). The total effect remained significant (p = .018), and approximately 67% of the effect of right SFG activity on INQ was mediated through depressive symptoms (proportion mediated = 0.67, p = .024) (**Figure 4**).

## 4 Discussion

This study investigated the electrophysiological and psychological characteristics of adolescents engaging in non-suicidal self-injury (NSSI), focusing on no-go P3 amplitude and its relationship with impulsivity and interpersonal distress. Our key findings were: (1) the NSSI group showed significantly lower accuracy in the go/nogo task, (2) the NSSI group showed reduced no-go P3 amplitude at Fz compared to those of the HC, (3) this reduction was associated with increased depressed symptoms and interpersonal distress, (4) source-level activity in the right superior frontal gyrus (SFG) was marginally lower in the NSSI group but diminished after adjusting for depressive symptoms, (5) this reduction was also associated with increased depression and interpersonal distress, and (6) mediation analysis revealed that depressive symptoms significantly mediated the relationship between inhibitory neural markers and interpersonal distress.

### 4.1 Behavioral inhibition deficits in NSSI

The NSSI group showed poorer behavioral performance in both go and no-go trials, reflecting diminished recruitment of cognitive resources (40). In previous studies, no-go P3 has been shown to be related to impulsivity (15, 20, 41). Consistent with previous studies, the NSSI group exhibited reduced no-go P3 amplitudes at Fz electrodes compared to HC, suggesting impaired inhibitory control and reduced allocation of attentional resources in this population. This result also aligns with previous findings in which diminished P3 amplitudes during no-go tasks were associated with impaired impulse control (20, 41). However, it is noteworthy that after controlling for depressive symptoms, no significant group differences were found in self-reported measures of impulsivity (UPPS-P) or emotion regulation difficulties (DERS). These results suggest that the observed differences in neural markers may not fully correspond to self-perceived deficits in these domains, underscoring the need for cautious interpretation and further investigation into potential dissociations between subjective and neurophysiological indices. Notably, the reduction in no-go P3 amplitude was confined to the Fz site, with no significant group differences observed at other midline electrodes. This spatial specificity is consistent with prior electrophysiological studies indicating that response inhibition related P3 components are most robustly expressed over frontal midline regions, particularly Fz and FCz, which are closely associated with prefrontal executive functioning (42, 43).

### 4.2 Neural-affective interaction: P3 and interpersonal distress

In our study, no-go P3 amplitude at Fz was negatively correlated with both depressive symptoms and interpersonal distress, as measured by the INQ. These findings suggest that impaired inhibitory control and depressed mood frequently co-occur with interpersonal difficulties in adolescents engaging in NSSI. These findings are consistent with the IPTS, which posits that TB and PB, core elements captured by the INQ, are critical contributors not only to suicidal ideation, but also to self-injurious behavior (44, 45). Within this framework, reduced inhibitory capacity may impair the regulation of socially aversive impulses, thereby fostering feelings of interpersonal alienation (13). Together, our findings underscore a constellation of overlapping vulnerabilities—neurophysiological, affective, and social—that characterize NSSI, and point to the importance of integrated interventions targeting both inhibitory control and mood to alleviate interpersonal pain.

### 4.3 Source analysis and social-cognitive control

Although Bayes factors provided only weak evidence for group differences in right SFG activity (BF_10_ ≈ 1.38), source‐level findings enriched our ERP results by implicating higher‐order social–cognitive control mechanisms. The dorsal SFG, often grouped with dorsomedial prefrontal cortex, is a core node of the mentalizing network, has been implicated in top-down regulation of emotional interference and conflict, particularly in tasks requiring suppression of affective distractors and the resolution of socially salient cues (46–48). Recent neuroimaging studies in individuals with MDD have reported SFG dysfunction associated with emotion dysregulation and self-injurious behavior, raising the possibility that similar mechanisms may be present in adolescents with NSSI (48). In the present study, decreased right SFG activation was significantly associated with both depressive symptoms and interpersonal distress, and mediation analysis indicated that depressive symptoms accounted for the relationship between frontal hypoactivation and INQ scores. While the right SFG has not been consistently identified as a direct neural substrate of interpersonal distress, the reduced right SFG activation observed in adolescents with NSSI may reflect an upstream deficit in inhibitory control and depressive symptoms. Prior research has implicated the SFG in the modulation of emotional responses during interpersonal conflict and social competition (47, 49), suggesting that diminished engagement in this region may compromise the capacity to regulate affectively charged interpersonal interactions.

### 4.4 Depressive symptoms as a mediator of neural-social links

When we included depressive symptoms (CES-D) as a covariate in our scalp‐level RMANOVA, the Group × Electrode interaction for no-go P3 remained significant, indicating frontal‐midline inhibition deficits in NSSI beyond mood effects. In contrast, applying the same covariate adjustment to our source‐level analyses abolished all group differences in right SFG activity. This pattern suggests that source-localized activity largely reflects depressive severity.

Crucially, because no-go P3 amplitude at Fz correlated with both depressive symptoms and interpersonal distress, we pursued a mediation model in which depressive symptoms bridge the neural inhibition (no-go P3 amplitude and right SFG activation) and interpersonal distress. Mediation analysis revealed that depressive symptoms significantly mediated the association between diminished no-go P3 amplitude, Rt. SFG activation and interpersonal distress. This result supports the interpretation that inhibitory deficits may contribute to social distress primarily through their impact on affective dysregulation (50).

### 4.5 Limitations

Our study had some limitations. First, due to the cross-sectional design, we could not assess causal relationships between neural responses and clinical features. Longitudinal studies are needed to clarify the temporal dynamics of these associations. Second, various scales for measuring clinical characteristics were evaluated using self-report measures. Despite the self-reported scales used in this study having good stability and validity, they could not reflect the neural or cognitive basis of clinical characteristics such as impulsivity and emotional regulation. Third, our results are limited to patients with MDD, requiring further studies on accompanying self-harm in other psychiatric diseases. Fourth, because our participants were adolescents, systematic assessments of stable personality traits (e.g., borderline or avoidant features) were not conducted. This may limit interpretation of trait-level contributors to interpersonal distress and inhibitory control.

### 4.6 Conclusions

In summary, adolescents engaging in NSSI exhibit co-occurring deficits in fronto-midline inhibitory control, elevated depressive symptoms, and heightened interpersonal distress, with depressive mood mediating the link between neural inhibition markers and social pain. These findings underscore the value of integrated interventions that simultaneously target cognitive control and mood regulation to alleviate interpersonal suffering and reduce self-injurious behaviors. Future longitudinal and experimental studies should test whether strengthening inhibitory capacity or alleviating depressive symptoms can disrupt this vulnerability cluster and inform tailored prevention and treatment strategies.

## Data Availability

The datasets presented in this article are not readily available because of ethical and privacy concerns. Requests to access the datasets should be directed to JK, ideal91@hanmail.net.
